# Cross-Border Circulation and Molecular Surveillance of HIV-1 in the Azov and Donbas Regions: A Study of Genetic Diversity and Drug Resistance

**DOI:** 10.3390/v18080856

**Published:** 2026-08-05

**Authors:** Anastasiia Antonova, Anatolii Vinokurov, Daria Kustova, Andrei Pochtovyi, Daria Ogarkova, Ruslan Adgamov, Anna Kuznetsova, Elena Tsyganova, Inna Kulikova, Andrei Plutnitskii, Vladimir Gushchin, Aleksandr Gintsburg, Denis Logunov, Aleksei Mazus

**Affiliations:** 1 Federal State Budget Institution National Research Center for Epidemiology and Microbiology Named After the Honorary Academician N.F. Gamaleya, The Ministry of Health of the Russian Federation, 123098 Moscow, Russia; 2 Department of Virology, Lomonosov Moscow State University, 119234 Moscow, Russia; 3 Department of Medical Genetics and Postgenomic Technologies, Sechenov First Moscow State Medical University, 119991 Moscow, Russia; 4 Moscow City Center for AIDS Prevention and Control, 105275 Moscow, Russia; 5 Department of Emergency Medical Assistance Organisation and Health Risk Management of the Ministry of Health of the Russian Federation, 127994 Moscow, Russia; 6 Federal Medical Biophysical Center Named After A.I. Burnazyan, 123098 Moscow, Russia; 7 Department of Infectiology and Virology, Sechenov First Moscow State Medical University, 119991 Moscow, Russia

**Keywords:** HIV-1, molecular epidemiology, dolutegravir resistance, molecular transmission networks, sub-subtype A6, Azov and Donbas regions

## Abstract

As critical geopolitical and migratory hubs in Eastern Europe, the Azov and Donbas regions represent an epidemiological melting pot for HIV-1 trafficking, exacerbating the global trend of rising dolutegravir (DTG) resistance through the cross-border dissemination of drug-resistant strains. This study presents a comprehensive molecular epidemiological analysis of HIV-1 in these regions in 2025 (N = 1666), focusing on drug resistance and cross-border transmission networks using phylogenetic and molecular network approaches. The study cohort was predominantly female (53.33%) and had heterosexual transmission (78.77%). Most patients (87.64%) received antiretroviral therapy (ART). Sub-subtype A6 predominated, with the radiation’s origin traced to September 1994. Molecular network analysis identified the study area as a significant node, demonstrating viral exports towards the Russian Federation and Belarus, alongside multiple imports from Ukraine, Poland, and Russia. The overall resistance prevalence was 4.49% to integrase strand transfer inhibitors (INSTIs), 2.49% to protease inhibitors (PIs), 12.19% to nucleoside reverse transcriptase inhibitors (NRTIs), and 16.07% to non-nucleoside reverse transcriptase inhibitors (NNRTIs). Surveillance drug resistance mutations in treatment-naive individuals stood at 0.89% (INSTIs), 4.90% (PIs), 4.90% (NRTIs), and 8.82% (NNRTIs). Crucially, intermediate or high-level DTG resistance and key mutations (G118R, R263K, and Y143R) were detected in individuals without prior DTG exposure. This 4.49% integrase inhibitor resistance cannot be considered low; combined with intense cross-border viral dissemination, it may indicate the formation of a stable pool of resistant variants, potentially posing a risk of dolutegravir-based regimen failure and highlighting the need for enhanced regional molecular surveillance.

## 1. Introduction

The disease caused by the human immunodeficiency virus (HIV) remains one of the most significant global public health threats. The development and spread of HIV drug resistance to antiretroviral therapy (ART) compounds this problem, destabilising socioeconomic development and leading to increased treatment costs and burden on national health systems.

The epidemic process in the Eastern Europe and Central Asia (EECA) region remains one of the most dynamic in the world, with the vast majority of infections still associated with the spread of the A6 genetic variant (previously classified as A1 former Soviet Union sub-subtype (FSU) subtype) [1]. Since its emergence in the mid-1990s, this variant has demonstrated exceptional evolutionary stability and rapid spread, initially among injection drug users (IDUs) and, over the last decade, via heterosexual transmission [2,3,4,5].

Border regions of Ukraine with increased migration activity played a special role in the spread of HIV infection [6]. Of particular interest are the territories of the Azov and Donbas regions—industrial and transport regions with a high population density and intensive migration flows, which historically link them to major epidemic foci in both Russia and Ukraine [6,7].

Beyond migration, recent geopolitical events have further influenced the HIV epidemiological situation in the EECA region and neighboring countries. For example, Europe is currently experiencing an increase in the number of HIV infections caused by migration flows from Ukraine and by the A6 sub-subtype [8].

The COVID-19 pandemic destabilised healthcare systems and disrupted HIV prevention and treatment programs in Ukraine. This, in turn, increased both new infections and the likelihood of drug resistance [9].

Consequently, due to intense migration and developed infrastructure, the Azov and Donbas regions serve as an active center for the formation of new transmission chains and a reservoir for the spread of resistant strains of HIV-1.

The aim of this study is to perform a comprehensive molecular epidemiological analysis of HIV-1 in the Azov and Donbas regions, with a primary focus on monitoring drug resistance to integrase inhibitors (INSTIs). Additionally, the study aims to reconstruct viral transmission routes and assess the dynamics of cross-border transmission cluster formation.

## 2. Materials and Methods

### 2.1. Study Cohort and Data Acquisition

The study utilised a consecutive sampling strategy to collect biological samples (plasma and whole blood) from unique HIV-positive individuals. These materials were collected during routine clinical visits at regional AIDS centres in the Azov and Donbas regions (Donetsk, Luhansk, and Zaporizhzhia regions) throughout 2025. Given that these healthcare institutions serve as the primary hubs for HIV care and medication distribution, the resulting cohort provides a representative cross-section of the local patient population regarding demographics and modes of infection.

Analysis focused on HIV-1 *pol* gene sequences, specifically the integrase (INT) (predominantly), protease, and reverse transcriptase (PRRT) coding regions, which were analysed alongside demographic, clinical and epidemiological metadata.

Ethical standards were maintained in accordance with the Declaration of Helsinki, with all participants providing informed consent and data being strictly anonymised through unique coding. In addition, the Ethics Committees of the N.F. Gamaleya National Research Center of Epidemiology and Microbiology (protocol No. 85, 18 December 2024) approved the study.

### 2.2. Study Design and Sample Selection

**Figure 1:**
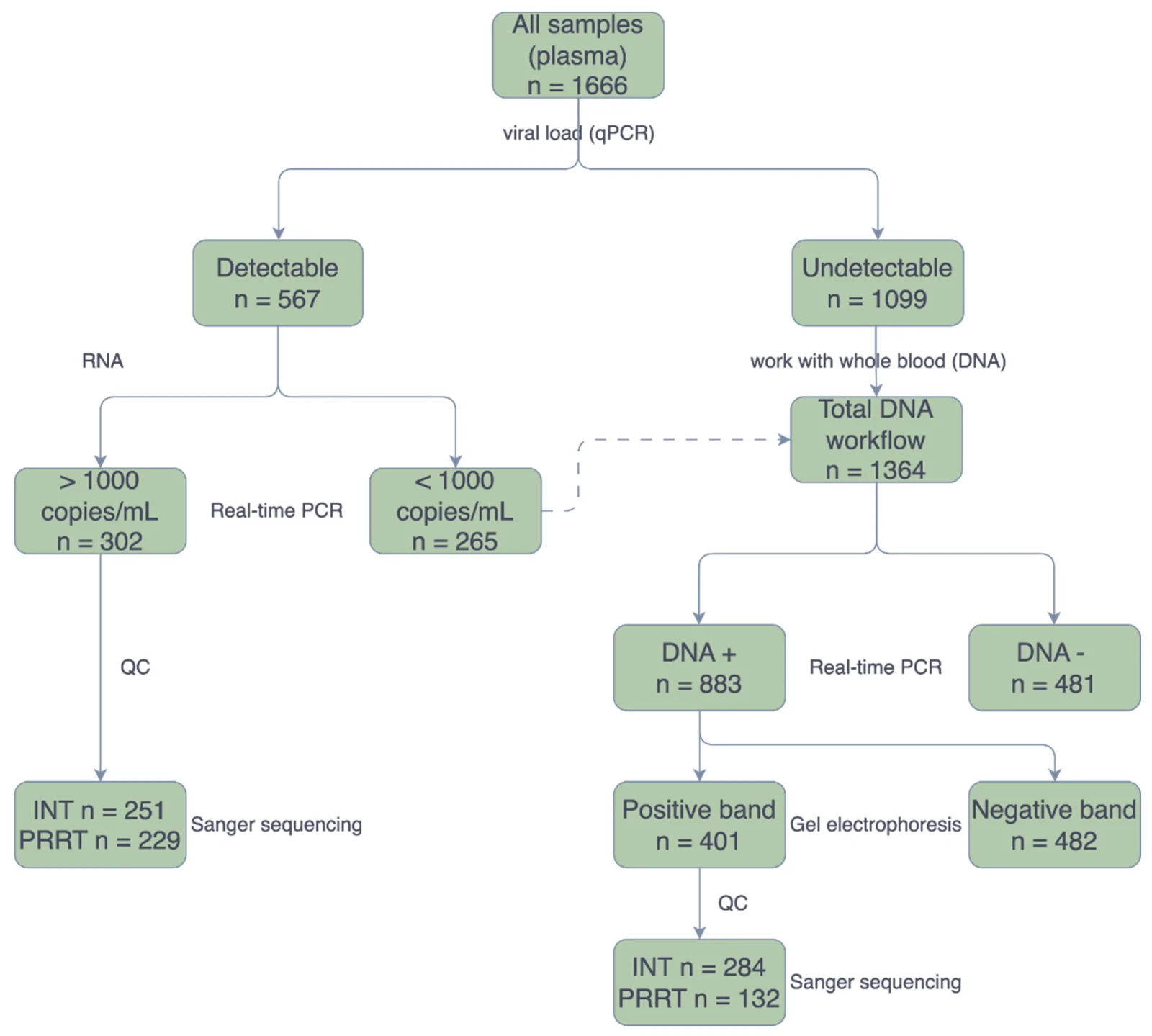
Flowchart of the study design and sample selection. *Note:* Approximately two-thirds of the initial 1666 plasma samples showed an undetectable viral load via RT-qPCR. This is primarily attributed to successful virological suppression driven by effective ART and high treatment adherence. Crucially, this widespread suppression, combined with the inclusion of low-level viraemia samples (<1000 copies/mL) into the DNA workflow, explains the observed 35.26% (481/1364) proviral DNA negativity rate. Under sustained virological suppression, the peripheral latent reservoir contracts significantly. Consequently, when utilising standard diagnostic sample volumes (100 µL of whole blood, yielding a threshold of 250 copies/mL), target proviral templates can fall below the analytical detection limits. This occurs due to stochastic sampling effects (the random absence of target DNA within the sample) rather than technical failure.

The comprehensive flowchart of the sample selection process is presented in Figure 1.

The initial screening pool of the study comprised 1666 biological samples (plasma). Based on viral load (VL) detection determined by quantitative real-time PCR (RT-qPCR) using the commercial “Vector-Best HIV-RNA Quantitative kit” (“Vector-Best”, Novosibirsk, Russia; sensitivity threshold of 11,6 copies/mL, a lower limit of quantification (LLoQ) of 50 copies/mL), these samples were first stratified into detectable and undetectable groups, and subsequently processed via two distinct workflows.

- *RNA workflow (Plasma)*: This pathway included samples with a detectable viral load (n = 567). The sub-cohort with high-level viraemia (>1000 copies/mL, n = 302) proceeded directly to downstream analysis. Conversely, samples with low-level viremia (<1000 copies/mL, n = 265) were rerouted to the DNA workflow.
- *DNA workflow (Whole blood)*: This pathway encompassed samples with an initially undetectable viral load (n = 1099) combined with those rerouted from the low-level RNA group, resulting in a total DNA workflow pool of 1364 samples. Target proviral DNA presence was verified via qualitative real-time PCR using the commercial “RealBest DNA HIV (WB) kit” (“Vector-Best”, Novosibirsk, Russia) strictly in accordance with the manufacturer’s protocol. DNA extraction was performed using a standard diagnostic initial sample volume of 100 µL of whole blood. The assay possesses an analytical sensitivity threshold of 25 copies per extracted sample volume, which mathematically establishes an effective concentration limit of detection at 250 copies/mL (25 copies/0.1 mL). Based on these operational parameters, the study population was stratified into primary screening-positive (DNA+, n = 883) and screening-negative (DNA−, n = 481) groups. The positive samples were further validated by gel electrophoresis; only the subgroup with a confirmed specific signal (positive band, n = 401) proceeded to sequencing, while the rest showed no specific amplification (negative band, n = 482).

All successfully amplified samples (n = 302 from the RNA workflow; n = 401 from the DNA workflow) underwent Sanger sequencing. The resulting sequences underwent stringent quality control (QC). We excluded low-quality sequences with atypical lengths, premature stop codons, major deletions, or frameshift mutations.

To analyse the viral genome, sequencing was performed for two distinct HIV-1 genomic regions following a stratified strategy.

- INT Region (Primary Focus): To thoroughly evaluate the prevalence of resistance to integrase inhibitors, and to dolutegravir (DTG) in particular, the molecular screening was prioritised towards the INT region. Consequently, successful amplification and sequencing were achieved for a total of 535 unique samples (n = 251 within the RNA workflow and n = 284 within the DNA workflow).
- PRRT region: This region was sequenced complementarily only for patients who had residual biomaterial available, thereby avoiding repeat blood collection (final sample sizes: n = 229 for RNA and n = 132 for DNA). Crucially, because the PRRT fragment is nearly twice as long as the INT region, providing significantly higher phylogenetic resolution, this dataset was specifically utilised for subsequent advanced analyses, including phylogenetic reconstruction and transmission cluster identification.

To bridge the gap between sequencing outputs and population-level analysis, data were consolidated at the patient level based on successful amplification and quality control. For the drug resistance analysis, datasets were constructed based on drug class relevance:

- PRRT Dataset (PIs, NRTIs, NNRTIs): The final analytical cohort comprised 361 unique patients, including 301 who yielded high-quality sequences for both PRRT and INT regions, and 60 with PRRT sequences only;
- INT Dataset (INSTIs): The final integrase analytical cohort comprised 535 unique patients. Among the total prospective cohort evaluated in the current study were the same 301 patients with both regions sequenced and 234 with INT sequences only.

### 2.3. HIV-1 Nucleic Acid Extraction and Sequencing

Clinical staff at regional AIDS centres performed procedures for the collection and fractionation of whole blood to isolate plasma. To extract viral RNA from plasma and proviral DNA from whole blood specimens, we utilised HiPure Viral RNA and HiPure Blood DNA Mini kits (Magen Biotechnology Co., Ltd., Guangzhou, China), respectively.

Sequencing of the HIV-1 *pol* gene, specifically the integrase (INT) coding region (spanning positions 4230–5096 bp relative to the HXB2 reference strain, GenBank K03455) and the protease-reverse transcriptase (PRRT) coding region (spanning positions 2253–3670 bp), was performed via Sanger-based in-house protocols.

### 2.4. HIV-1 Genotyping and Phylogenetic Analysis

Sequence alignment was performed using the ClustalW module within AliView v.1.27 [10], followed by manual correction where necessary to resolve problematic regions. The consensus alignment length was 1099 nucleotides (corresponding to positions 2253–3351 bp relative to the HXB2 reference strain, GenBank K03455) to cover all key HIV-1 drug resistance mutations. Although both the PRRT and INT regions were analysed in this study, the PRRT fragment was selected for genetic variant identification due to its greater length and its role as a well-established target commonly utilised in commercial genotyping assays.

Identification of HIV-1 genetic variants was performed using specialised online platforms, including COMET HIV-1 [11] and the HIVdb Program Sequence Analysis (v. 9.8) [12]. Samples with ambiguous subtyping results were further evaluated for potential recombination events using the jpHMM algorithm [13].

To validate the genotyping results, phylogenetic analysis incorporated reference sequences retrieved from the Los Alamos National Laboratory HIV Sequence Database (https://www.hiv.lanl.gov/content/sequence/NEWALIGN/align.html, accessed on 17 March 2026). Phylogenetic trees were constructed using the Maximum Likelihood (ML) method in IQ-TREE (v. 2.0.3) [14], employing the following parameters: -s [file] -m MFP -bb 1000. This approach included 1000 bootstrap replicates to assess branch support. The optimal nucleotide substitution model was automatically determined by the software, and clusters with bootstrap values ≥ 0.9 were defined as statistically significant. Final tree visualisation and annotation were performed using the Interactive Tree of Life (iTOL) software (v. 7) [15].

### 2.5. Phylodynamic Analysis and Identification of Transmission Clusters

To explore the evolutionary trajectory and transmission dynamics of the HIV-1 A6 sub-subtype, which is the most prevalent genetic variant in the region, phylodynamic analysis was performed using the Nextstrain pipeline (v. 10.4.2) [16].

The analysis dataset was constructed by integrating the newly sequenced A6-samples from this study (n = 352) with high-similarity reference sequences. To ensure a robust phylogenetic context, BLAST (v. 2.2.30) searches (https://www.hiv.lanl.gov/content/sequence/BASIC_BLAST/basic_blast.html, accessed on 3 April 2026) were performed for each study sequence, retrieving up to 1000 global sequences per query based on a similarity threshold of ≥97%. This threshold integrated both direct epidemiological links and more distant ancestral lineages necessary for stable molecular clock calibration.

After excluding duplicates and poor-quality sequences, the refined dataset was processed using the Nextstrain CLI toolset (v. 10.4.2). To reduce sampling bias, a multi-stage subsampling strategy in the Augur pipeline normalised the dataset to a maximum of 40 sequences per geographical unit per year. Representative sequences from various countries (e.g., Austria, Japan) were retained due to their unique phylogenetic proximity to the identified clusters, serving as essential markers for verifying transboundary spread.

Sequences were aligned using MAFFT, followed by Maximum Likelihood (ML) tree construction. Temporal scaling and ancestral state reconstruction were executed via TreeTime. To validate the temporal signal, a root-to-tip regression analysis was conducted, with tree rerooting performed using the least-squares method. This approach reconstructed the spatial and temporal viral spread within the Azov and Donbas regions, allowing visualisation of lineage-specific transmission clusters and their divergence via the Auspice platform (v. 2.66.0).

### 2.6. Molecular Transmission Network Analysis

To obtain a detailed overview of the regional epidemic process, molecular transmission network analysis covered all identified HIV-1 genetic variants. This approach evaluated transmission patterns beyond the dominant A6 sub-subtype, enabling the detection of potential infection chains within less prevalent groups, such as CRF63_02A6, subtype B and CRF03_A6B. Such an analysis is critical for identifying active transmission clusters within the Azov and Donbas regions during the current period (2023–2025) and for monitoring novel viral introductions.

The analysis was conducted using the MicrobeTrace platform (CDC, Atlanta, GA, USA). Unlike traditional phylogenetic reconstruction, this network-based approach utilises pairwise genetic distances to identify direct relationships (links) between sequences. Transmission clusters were defined using the Tamura-Nei (TN93) substitution model with a stringent genetic distance threshold of 0.005 (0.5%) substitutions per site to identify recent transmission events. This methodology identified active molecular clusters even within minor genetic variants.

The study dataset included the newly sequenced samples (n = 361) with closely related BLAST reference sequences. To ensure high resolution, specific similarity thresholds were applied: ≥95% for subtype B and CRF03_A6B, and ≥97% for A6 and CRF63_02A6. Up to 1000 matches per study sequence were initially retrieved; after removing duplicates and poor-quality reads, a final dataset of 2878 sequences was established for network reconstruction. A comprehensive metadata file, including gender, transmission route, collection year, and country, was integrated to characterise the socio-demographic drivers of HIV-1 spread.

### 2.7. Assessment of HIV-1 Drug Resistance Mutations

Drug resistance mutations (DRMs) affecting protease inhibitors (PIs), nucleoside reverse transcriptase inhibitors (NRTIs), and non-nucleoside reverse transcriptase inhibitors (NNRTIs) were identified using the Sierra algorithm within the HIV Drug Resistance Database (HIVdb Programme, v. 9.8). To characterise and interpret levels of drug resistance (DR) and surveillance drug-resistance mutations (SDRMs), we utilised the Mutations Analysis and the Calibrated Population Resistance (CPR) tools. The latter was specifically used to assess primary drug resistance (PrimDR) among antiretroviral therapy (ART)-naive individuals. All database queries were performed on 3 April 2026.

Resistance levels were categorised based on the Penalty Score into five levels: susceptible (score 0–9), potential low-level resistance (10–14), low-level resistance (15–29), intermediate resistance (30–59), and high-level resistance (≥60). Sequences with a score ≥15 were classified as resistant. From a clinical perspective, low-level resistance suggests that the drug may still retain activity but carries a significantly increased risk of rapid further mutation accumulation under continued selective pressure. Intermediate resistance implies a substantial reduction in virological response unless the drug is paired with a fully active background regimen, whereas high-level resistance indicates a near-total loss of drug efficacy, signifying definitive regimen failure and the urgent necessity for an optimised salvage therapy.

This classification was applied to the following antiretroviral agents:

*INSTIs:* bictegravir (BIC), cabotegravir (CAB), dolutegravir (DTG), elvitegravir (EVG) and raltegravir (RAL);

*PIs:* atazanavir (ATV), darunavir (DRV), indinavir (IDV), lopinavir (LPV), fosamprenavir (FPV), nelfinavir (NFV), saquinavir (SQV), and tipranavir (TPV);

*NRTIs:* zidovudine (AZT), stavudine (D4T), didanosine (DDI), emtricitabine/lamivudine (FTC/3TC), and tenofovir (TDF);

*NNRTIs:* dapivirine (DPV), efavirenz (EFV), etravirine (ETR), nevirapine (NVP), and rilpivirine (RPV).

### 2.8. Statistical Analysis

Statistical analysis utilised the R programming language (v. 4.2.2; R Foundation for Statistical Computing, Vienna, Austria) via the RStudio interface (v. 2023.03.0 + 386; Posit Software, PBC, Boston, MA, USA), alongside STATISTICA software (v. 6.0; StatSoft, Tulsa, OK, USA).

Categorical variables were summarised as frequencies and percentages. The prevalence of drug resistance mutations (DRMs) was calculated alongside 95% confidence intervals (CIs). Comparative analysis of these data was conducted using Pearson’s chi-squared test (ꭓ^2^); in instances where expected cell frequencies were low, Yates’s continuity correction or Fisher’s exact test (two-tailed) was applied as appropriate. Statistical significance was defined at a threshold of *p* < 0.05. All data visualisations were generated within the R environment.

## 3. Results

### 3.1. Study Cohort and Data Acquisition

A comprehensive analysis of demographic, clinical, and epidemiological metadata was conducted for the entire study cohort, including 1666 unique patients with HIV infection in the Azov and Donbas regions in 2025.

Regarding the gender distribution within the cohort (available for 1652 patients), females predominated, accounting for 53.33% of cases (n = 881; 95% CI: 50.92–55.72), whereas males constituted 46.67% (n = 771; 95% CI: 44.28–49.08). Demographic analysis of age characteristics (available for 1634 participants) indicated a median age of 41 years, with the interquartile range (IQR): 38–51 years.

The primary route of HIV infection transmission was successfully determined for 1583 individuals, while data were missing or not applicable (NA) for 83 patients. The absolute predominant route of HIV-1 transmission was heterosexual contact, accounting for 78.77% of cases (1247/1583; 95% CI: 76.69–80.72). Injecting drug use (IDU) represented the second most frequent transmission route, recorded in 16.99% of individuals (269/1583; 95% CI: 15.22–18.92). Vertical (mother-to-child) transmission was documented in 3.22% of cases (51/1583; 95% CI: 2.45–4.22), while homosexual contact (MSM) was markedly less frequent, representing only 0.44% (7/1583; 95% CI: 0.19–0.93). Other specific epidemiological contexts—including cases with unidentified close contact, documented contact of damaged mucosal or wound surfaces, and transmissions associated with invasive medical procedures—collectively accounted for 0.57% of the stratified cohort (9/1583; 95% CI: 0.28–1.09).

Clinical data on ART status were available for 1626 of the 1666 patients. Within this group, the vast majority (n = 1444; 88.81%, 95% CI: 87.18–90.25) were receiving ART, while 182 individuals (11.19%, 95% CI: 9.75–12.82) were treatment-naive. The ART status of the remaining 40 patients could not be clearly determined. The most prevalent therapeutic regimens utilised within the study group were TDF + 3TC + EFV and TDF + 3TC + DTG.

Immuno-virological profiles revealed significant differences between these two study groups. Among the treatment-naive individuals, baseline CD4+ T-cell count data were available for 133 patients, yielding a median value of 291 cells/μL (IQR: 119–538.5 cells/μL). Virological assessment of this subgroup (n = 151 with quantifiable data) showed a median plasma HIV-1 RNA viral load of 3.94 log_10_ copies/mL (IQR: 3.12–4.77 log_10_ copies/mL). In contrast, patients receiving ART (with available immunological data n = 1416) showed robust immune reconstitution, with a median CD4+ T-cell count of 529.5 cells/μL (IQR: 342.0–737.0 cells/μL). Active viral replication was quantified in only 271 ART-experienced individuals, with a median viral load of 3.34 log_10_ copies/mL (IQR: 2.34–4.34 log_10_ copies/mL); the vast majority of treated patients (n = 1171) achieved complete virological suppression.

### 3.2. HIV-1 Subtyping and Phylogenetic Analysis

Integrating online subtyping tools and phylogenetic analysis revealed a marked predominance of the A6 sub-subtype, which accounted for 97.51% (352/361; 95% CI: 95.32–98.85) of the analysed sequences.

The rest of the cohort included less prevalent lineages, such as CRF63_02A6 in 1.66% of cases (6/361; 95% CI: 0.61–3.58), and subtype B, representing 0.55% (2/361; 95% CI: 0.07–1.99). Additionally, a single case of the recombinant form CRF03_A6B was detected, accounting for 0.28% (1/361; 95% CI: 0.001–1.53) of the total study participants. Comprehensive phylogenetic analysis confirmed these subtyping results (Figure S1).

### 3.3. HIV-1 Transmission Chains

**Figure 2:**
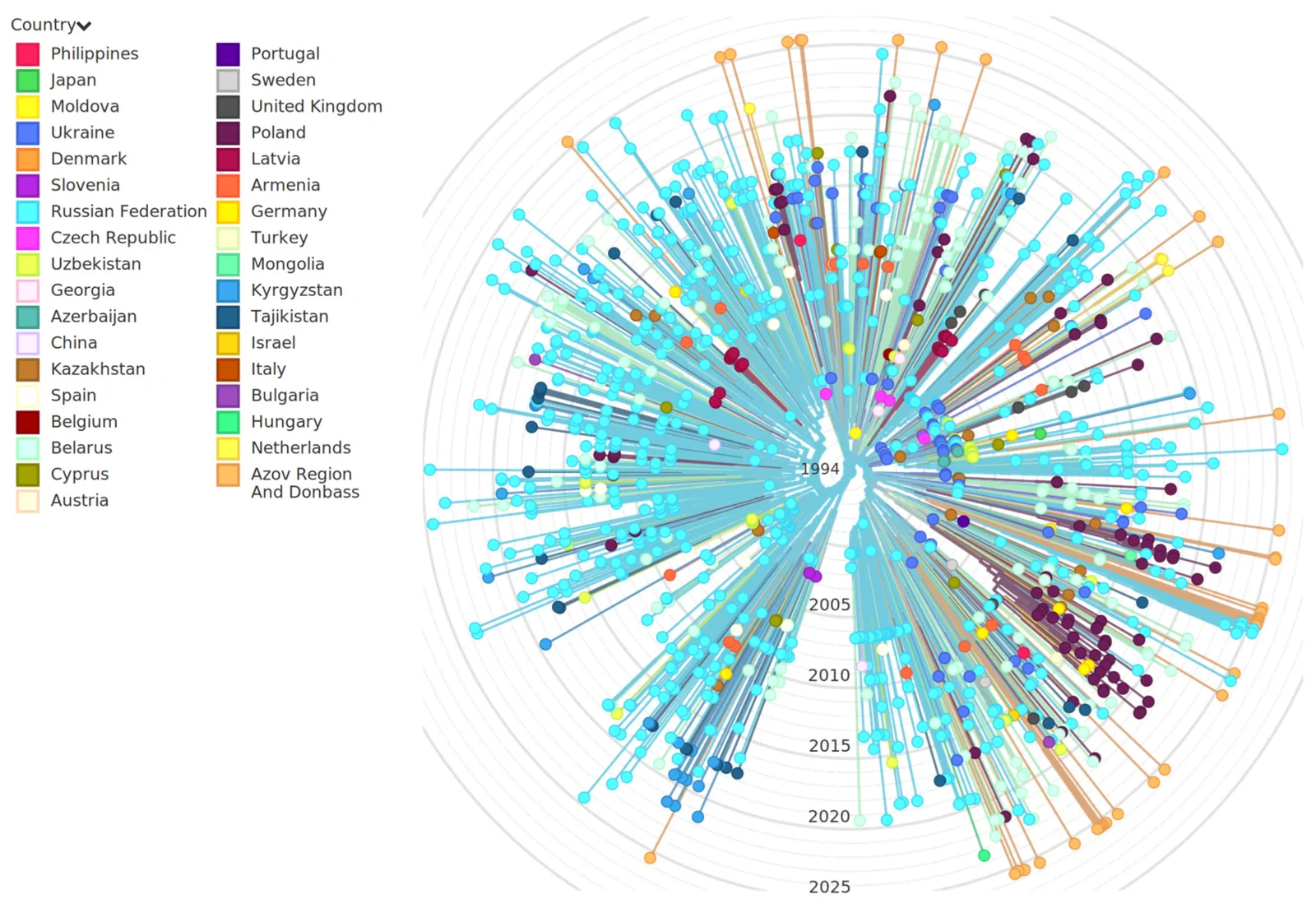
Maximum-likelihood phylogeny of A6-sequences from the Azov and Donbas regions with publicly available sequences from FSU and European countries.

The phylodynamic analysis of the HIV-1 A6 sub-subtype provided a high-resolution reconstruction of the viral evolutionary trajectory within the Azov and Donbas regions. The radial phylogenetic tree (Figure 2) identifies the time to the most recent common ancestor (tMRCA) of the regional radiation as of September 1994 (95% CI: February 1992–October 1995).

This origin aligns with the historical emergence of the A6 sub-subtype across the post-Soviet space [7]. The evolutionary rate was estimated at 1.42 × 10^−4^ substitutions per site per year, reflecting the molecular clock of the *pol* gene region. The estimated evolutionary rate reflects the high genetic conservation within the analysed dataset. This rate is attributed to the selection of closely related sequences using BLAST, focusing on stable transmission lineages within the A6 sub-subtype.

**Figure 3:**
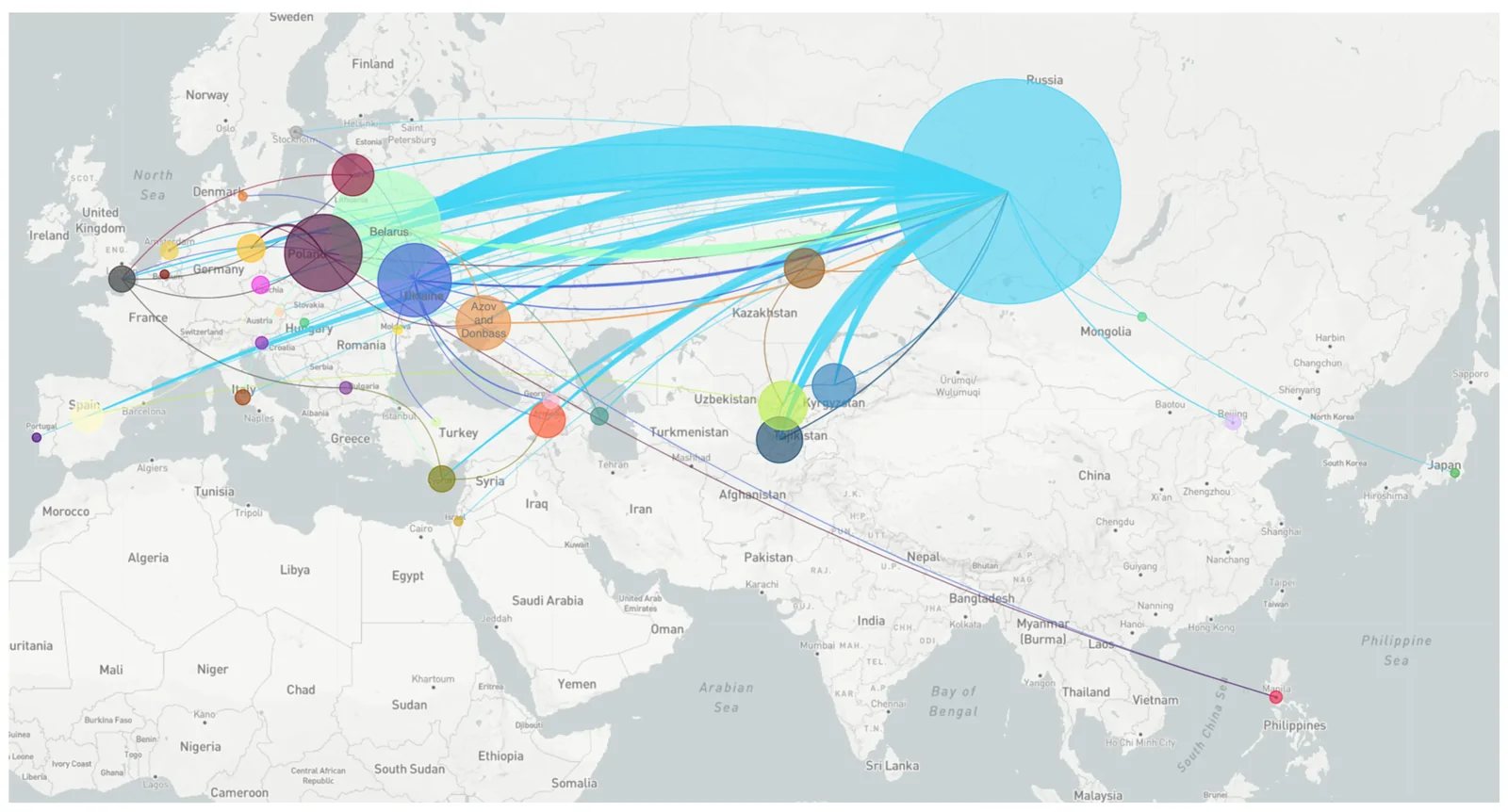
Geographical transmission routes of A6-sequences from the Azov and Donbas regions with publicly available sequences from FSU and European countries. The indicated transmission routes represent country-to-country movement rather than specific city-level localities.

The spatiotemporal reconstruction revealed a complex network of viral migration across the former Soviet Union (Figure 3).

The analysis highlights the Azov and Donbas regions as significant nodes within this network. Specifically, the data indicate strong genetic links between strains from the study area and sequences collected in the Russian Federation and Belarus. Conversely, multiple potential introduction events were identified, showing close genetic relationships with lineages originating from Ukraine, Poland and other regions of Russia. This multi-directional genetic exchange underscores the role of the study area as a dynamic transit hub where diverse viral lineages intersect. While the map illustrates extensive international connectivity, the visual dominance of certain links may be influenced by sampling bias, as genomic data availability varies across the neighbouring territories.

**Figure 4:**
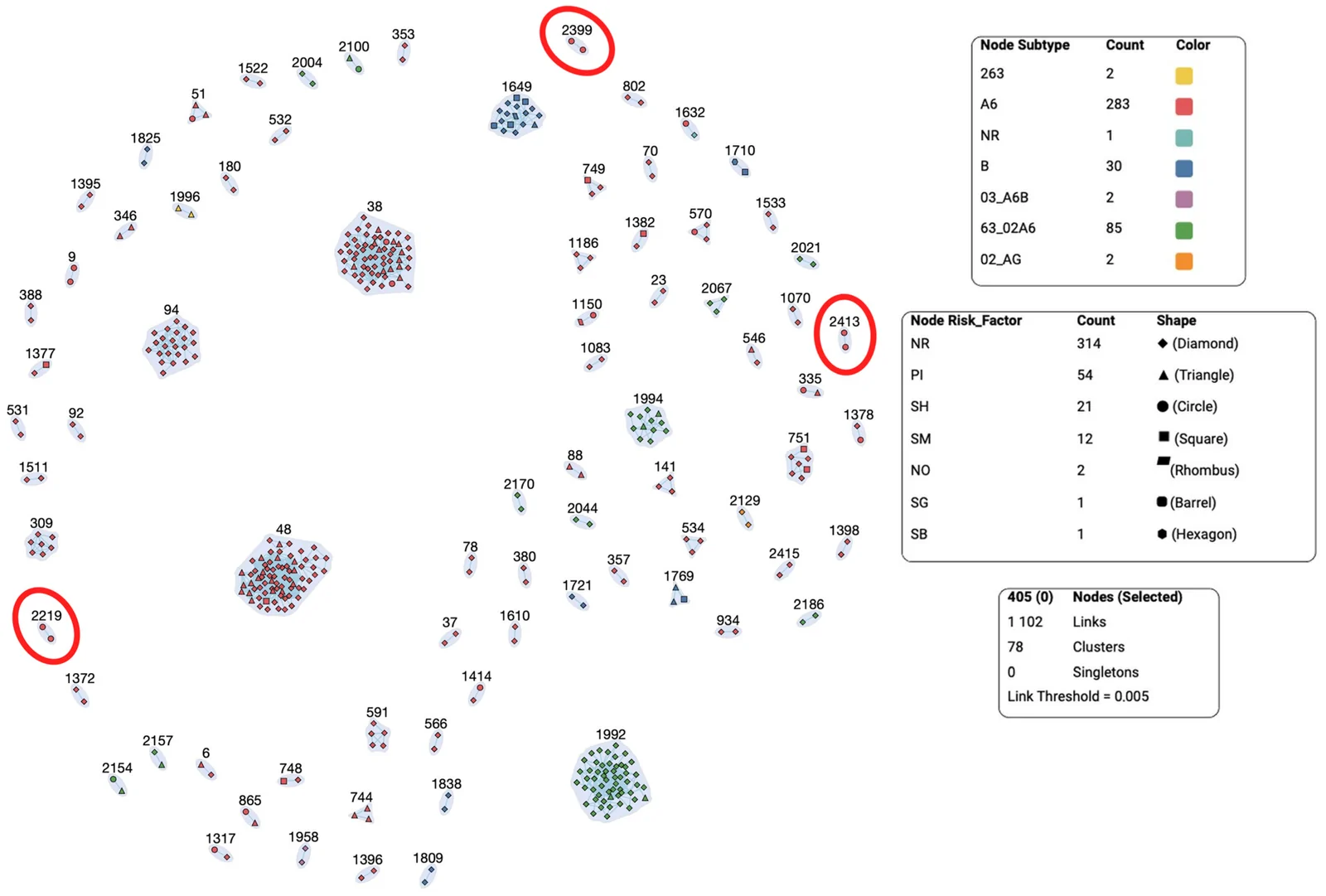
Network clustering results for the HIV-infected persons’ sequences (A6, B, CRF03 and CRF63 genetic variants) in the Azov and Donbas regions generated by MicrobeTrace (v.2.2). Red circles highlight significant transmission clusters that encompass sequence samples from the investigated study cohort. Due to unalterable system limitations of the software, the numeric value "0,005" is displayed with a comma, representing a decimal value of 0.005.

To evaluate the intensity of recent local transmission, a network analysis was performed using MicrobeTrace at a genetic distance threshold of 0.005 substitutions per site. The resulting transmission graph showed a highly fragmented topology (Figure 4).

Only three (№ 2219, 2399, 2413) small clusters, each consisting of two individuals (dyads), were identified within the study cohort. All individuals within these dyads reported heterosexual contact as the primary risk factor. The absence of large, complex clusters or “star-like” networks suggests a lack of active, high-intensity local outbreaks or concentrated transmission within specific social networks in the Azov and Donbas regions during the study period.

### 3.4. Assessment of HIV-1 Drug Resistance Mutations

The overall prevalence of resistance in the study cohorts was: 4.49% (24/535; 95% CI: 3.00–6.62) to INSTIs; 2.49% (9/361; 95% CI: 1.24–4.74) to PIs; 12.19% (44/361; 95% CI: 9.18–15.99) to NRTIs; and 16.07% (58/361; 95% CI: 12.62–20.23) to NNRTIs.

The prevalence of transmitted drug resistance (SDRMs) within the treatment-naive subcohorts was: 0.89% (1/112; 95% CI: 0.01–5.38) to INSTIs; 4.90% (5/102; 95% CI: 1.83–11.25) to PIs; 4.90% (5/102; 95% CI: 1.83–11.25) to NRTIs; and 8.82% (9/102; 95% CI: 4.52–16.11) to NNRTIs. A comprehensive, cross-tabulated breakdown of these prevalence rates stratified side-by-side by drug class and ART exposure status is provided in Table 1.

A high diversity was observed in the composition of resistance mutations to INSTIs, represented by major mutations: T66A, E92G/Q, G118R, E138K, G140R, Y143C/H/R/S, Q148R and R263K. Among the additional mutations identified, the L74I polymorphism was the most prevalent, occurring in 506 patients (94.58%; 95% CI: 92.30–96.23). Over 92% of the viral isolates demonstrated full susceptibility to the INSTI class overall, with the notable exception of CAB, to which only 4.67% (25/535; 95% CI: 3.16–6.84) of isolates retained full susceptibility. Specifically, 0.93% (5/535; 95% CI: 0.33–2.24) of isolates exhibited reduced susceptibility to DTG to varying degrees, including cases of intermediate and high-level resistance. Notably, among patients with intermediate or high-level DTG resistance, only one had a documented history of prior exposure to this drug, suggesting the transmission of cross-resistant variants within the study population. Among the drugs tested, CAB and RAL accounted for the highest frequency of high-level resistance cases—2.43% (13/535; 95% CI: 1.38–4.15) and 1.87% (10/535; 95% CI: 0.97–3.45), respectively.

The distribution of the most prevalent major drug resistance mutations (DRMs) across the studied antiretroviral classes is summarised in Table 2. Within the PI class, V82A was the most frequent mutation. NRTI resistance was predominantly driven by high frequencies of M184V and K65R, while NNRTIs revealed a clear predominance of the G190S substitution, closely followed by K101E, K103N, and Y181C mutations.

Notably, dual-class resistance involving both NRTIs and NNRTIs was identified in four treatment-naive individuals (3.92%; 4/102; 95% CI: 1.22–9.97), who exhibited complex and extensive mutational profiles. Specifically, three patients harboured highly complex substitution patterns: the first presented with A62V + K65R + M184V combined with K101E + Y181C + G190S; the second displayed K65R + S68G + K70T + M184V combined with K101N + V106I + V108I + E138G; and the third exhibited a robust multi-drug resistant backbone consisting of D67G + K70E + M184V coupled with K101E + G190S. The final case harboured the M184I substitution in combination with the E138K mutation.

Drug resistance profiles varied significantly across different antiretroviral classes (Figure S2). Viral isolates demonstrated the highest full susceptibility to most PIs, with rates exceeding 95%, except for minor reductions observed in FPV and NFV. In contrast, high susceptibility to NRTIs was less frequent; only AZT maintained a susceptibility rate above 95%, while the remaining agents showed lower frequencies of full susceptibility, with the highest rates of high-level resistance documented for FTC/3TC and DDI. The lowest susceptibility rates were observed for NNRTIs, where fewer than 85% of patients were susceptible to any drug in this class. Notably, high-level resistance was more pronounced among first-generation NNRTIs—NVP and EFV—compared to next-generation agents—RPV and DPV. Detailed quantitative data, including specific percentages, sample fractions, and 95% confidence intervals for all levels of resistance, are provided in the caption of Figure S2.

## 4. Discussion

A comprehensive analysis of HIV-1 in the Azov and Donbas regions indicates the persistence of the historically dominant A6 genetic variant. This variant became established among people who inject drugs (IDUs) in southern Ukraine in the mid-1990s, subsequently spreading rapidly throughout the post-Soviet space [1,2,3,17,18]. Our data correlate with phylodynamic studies indicating that large industrial centres, such as Donetsk and Luhansk, have historically served as key epidemiological hubs, facilitating regional dissemination of the virus to adjacent territories [6]. Currently, these regions continue to function as important transit hubs characterised by active, highly interconnected viral circulation, as reflected in the close genetic affinity of local strains with sequences from Ukraine, Poland, Belarus, and various regions of Russia. These migration processes, intensified by current geopolitical events, highlight the necessity for continuous molecular monitoring to control the spread of HIV infection. Notably, molecular cluster analysis revealed only a small number of dyads, indicating the current absence of large-scale local outbreaks and a predominantly fragmented pattern of virus transmission.

The current socio-demographic profile of patients in these regions is characterised by a predominance of heterosexual transmission over the parenteral (injection) route. Homosexual transmission (MSM) was documented in 0.44% of cases. This low proportion aligns with the cohort demographics, where a predominance of females mathematically limits the expected share of MSM lineages. However, strictly within the male subgroup (n = 771), the proportion of MSM transmission rises to 0.91% (7/771). Additionally, a potential under-reporting during retrospective clinical data collection cannot be ruled out, as patients may sometimes provide incomplete personal histories regarding sensitive transmission routes. Within the analysed cohort, females constitute the majority of patients (53.33% vs. 46.67% males). This gender imbalance closely mirrors the underlying demographic structure of the local population; while the average female proportion across the Russian Federation stands at 53.5%, recent spatial and demographic analyses indicate that this figure escalates to 55–58% in the studied territories [19]. The median age of the patients is 41 years, which underscores the ongoing stabilisation and general “ageing” trend of the HIV-infected population in these territories, consistent with broader epidemiological patterns observed elsewhere [20,21].

The drug resistance profile deserves particular attention regarding the effectiveness of antiretroviral therapy. The prevalence of transmitted drug resistance reached 8.82% for certain drug classes, falling within the moderate prevalence category (5–10%) according to the World Health Organization criteria [22,23]. However, a detailed analysis of the overall prevalence of resistance and individual mutations across different drug classes revealed several areas of concern. The most favourable situation was observed for protease inhibitors (PIs), where over 95% of patients demonstrated full susceptibility to most drugs, with the exception of FPV and NFV. Conversely, the situation was more alarming for NRTIs: high susceptibility was retained only for AZT (97.51% of patients), while it dropped to 91.97% and 88.37% for TDF and FTC/3TC, respectively. The most frequent mutations were M184V (7.48%) and K65R (5.54%), both of which reduce susceptibility to lamivudine/emtricitabine and tenofovir—the drugs forming the basis of standard first-line ART regimens [24,25,26]. It is well established that the M184V mutation is selected by 3TC/FTC, reducing susceptibility to these drugs more than 200-fold, while the K65R mutation reduces susceptibility 5–10-fold [27,28,29,30,31,32]. Consequently, the transmission of these resistant variants may significantly compromise the effectiveness of standard regimens.

The most difficult situation was observed for NNRTIs, with less than 85% of patients susceptible to each drug in the class. High-level resistance to EFV was detected in 12.19% of patients, with the G190S mutation prevalence reaching 6.65%. This mutation is the most common NNRTI-resistance mutation among individuals infected with sub-subtype A6 in the former Soviet Union; it requires only a single nucleotide substitution from the wild-type virus to develop and reduces EFV susceptibility more than 50-fold [33,34]. Combined with active migration flows, viral transmission to Russia, and the inclusion of EFV in first-line therapy in the Russian Federation [26], there is a significant risk of spreading resistant forms.

A stratified analysis linked these NRTI and NNRTI mutations to treatment histories and specific antiretroviral regimens. The vast majority of these mutations (approximately 70–82%) occurred in the ART-experienced subgroup. Notably, the standard first-line regimen (TDF + 3TC/FTC + EFV) predominated, accounting for over 50% of resistance cases, alongside historical regimens such as AZT + 3TC + EFV and ddI + 3TC + EFV. Virological profiles also clearly correlated with resistance status: the mean viral load exceeded 3.94 log_10_ copies/mL among treatment-naive individuals, and exceeded 3.34 log_10_ copies/mL among the ART-experienced patients, indicating a profound failure of viral suppression.

The dynamics of HIV-1 resistance to integrase strand transfer inhibitors (INSTIs) in the study regions demonstrated a statistically significant increasing trend (*p* < 0.0013, Fisher’s exact test). To ensure data integrity, a cohort-wide audit of longitudinal medical records traced complete treatment histories for all 535 patients from their initial HIV diagnosis. A previous 2019 study showed fully preserved susceptibility to INSTIs, with no mutations detected (0/190; 0.0%), coinciding with maximum efficacy of DTG-based regimens [35].

However, according to the World Health Organization, the overall prevalence of drug resistance to INSTIs, particularly to DTG, in Ukraine reached 3.7% during 2020–2021 [36]. Consequently, the emergence and spread of INSTI resistance require close monitoring, particularly in countries scaling up DTG as first-line therapy. In the present study, the observed resistance rate of 4.49% (24/535; 95% CI: 3.00–6.62) further confirms the rapid overcoming of the genetic barrier by modern INSTIs in the study regions. Given the strategic importance of INSTIs, this trend suggests a risk of the spread of resistance mutations.

A detailed analysis of resistance patterns among the 7 patients harbouring INSTI mutations revealed critical insights. Among the 4 patients carrying major dolutegravir (DTG)-associated mutations, 1 possessed the G118R mutation and 3 carried the R263K mutation. Applying the clinical definitions introduced in our methods, these specific variants represented the most clinically significant mutations within the cohort, conferring substantial resistance to second-generation inhibitors. The patient with the G118R mutation exhibited high-level resistance to DTG. From a clinical perspective, this indicates a near-total loss of drug efficacy, signifying definitive regimen failure and requiring an immediate transition to a new, fully active salvage regimen. Conversely, the 3 patients with the R263K mutation developed intermediate-level resistance. Clinically, this implies a substantial reduction in the virological response to DTG; however, the drug may still retain residual activity provided it is paired with a potent and fully active background regimen, though it demands heightened viral load monitoring. Crucially, all 4 individuals had been treated strictly with efavirenz (EFV)-based regimens and had no prior clinical exposure to any INSTIs (though 1 patient was subsequently switched to bictegravir). Because the cohort-wide chart reviews ruled out incomplete histories, the presence of these severe DTG-specific mutations in INSTI-naive individuals provides definitive evidence of true transmitted drug resistance (TDR). In contrast, the remaining 3 patients exhibited the Y143R mutation. For 2 of these individuals, the audited records confirmed a history of raltegravir (RAL), demonstrating a classic case of archived cross-resistance selected by first-generation INSTIs. The third patient, initially treated with EFV and subsequently optimised to a DTG-based regimen, most likely represents a case of acquired drug resistance (ADR) emerging under selective antiretroviral pressure. Overall, this granular patient-level data confirms that the observed resistance is driven by a complex interplay of both transmitted and acquired drug resistance within the population.

The rapid escalation of INSTI resistance from 0% [35] to 4.49% within just a five-year period demonstrates an alarming trajectory that can no longer be interpreted as a low or negligible indicator. This sharp upward trend serves as an urgent threshold signal that demands adjustments in both public health policy and routine clinical care in the region. Regarding healthcare policy, this dynamic justifies establishing systematic, routine regional surveillance of INSTI mutations. In clinical practice, these findings imply that clinicians must no longer assume DTG-based regimens are infallible. In cases of unexplained virological failure on DTG, physicians should not automatically attribute it to poor patient adherence alone; instead, early access to genotyping must be prioritised to promptly optimise salvage therapy and preserve future treatment options.

Strengths and Limitations. A major operational strength of this study is that all participating regional AIDS Centres operated continuously in a routine, standard mode, ensuring a reliable sampling framework from cohorts actively engaging with specialised care. However, several limitations must be noted. First, the observed gender distribution (53.33% females vs. 46.67% males) accurately mirrors the underlying population structure of these territories, where recent demographic analyses indicate the female proportion escalates to 55–58%; nevertheless, this imbalance inherently impacts demographic metrics by mathematically diluting the total proportion of homosexual transmission (MSM), which rises to 0.91% (7/771) when evaluated strictly within the male subgroup. Second, potential under-reporting due to incomplete personal histories remains an expected limitation inherent to retrospective registry data. Third, the sample size for drug resistance analysis was limited. Approximately two-thirds of the cohort achieved undetectable viral loads on successful ART, making PCR amplification technically impossible despite using highly sensitive nested assays. To optimise future molecular surveillance without altering routine testing protocols or requiring additional equipment, public health efforts should focus on pragmatic digital data integration to automate the linkage between existing clinical registries and laboratory databases within key centres. Ultimately, since these regional centres continue to operate fully in their standard mode, the continuation of consistent, long-term molecular monitoring remains highly realistic. This monitoring is essential to evaluate epidemiological indicators over time and assess targeted public health countermeasures.

## 5. Conclusions

The findings of this study necessitate further comprehensive monitoring in these regions, as well as the expansion of surveillance to adjacent border areas, where the probability of HIV-1 transmission, including drug-resistant variants, is markedly elevated.

The observed 4.49% prevalence of resistance to modern antiretroviral agents (INSTIs) cannot be considered negligible, given its rapid escalation from baseline levels over a five-year period. Combined with transmitted resistance and major mutation accumulation, these data indicate a potential expansion of the resistant HIV-1 reservoir. This trend poses a significant long-term risk to the durability and effectiveness of first-line DTG-based regimens in the region, thereby underscoring the urgent need for enhanced virological monitoring and proactive clinical vigilance.

## Figures and Tables

**Table 1:** Prevalence of drug resistance mutations stratified by antiretroviral class and treatment history.

Antiretroviral Class	Overall Prevalence *, % (n/N) [95% CI]	Transmitted Resistance (Naive), % (n/N) [95% CI]	Acquired/Archived (Experienced), % (n/N) [95% CI]
INSTIs	4.49%	0.89%	5.56%
	(24/535)	(1/112)	(22/396)
	[3.00–6.62]	[0.01–5.38]	[3.66–8.31
PIs	2.49%	4.90%	1.69%
	(9/361)	(5/102)	(4/237)
	[1.24–4.74]	[1.83–11.25]	[0.50–4.41]
NRTIs	12.19%	4.90%	15.19%
	(44/361)	(5/102)	(36/237)
	[9.18–15.99]	[1.83–11.25]	[11.15–20.35]
NNRTIs	16.07%	8.82%	17.72%
	(58/361)	(9/102)	(42/237)
	[12.62–20.23]	[4.52–16.11]	[13.36–23.11]

* Note: The total study population (N = 535 for INSTIs; N = 361 for other drug classes) encompasses treatment-naïve individuals, ART-experienced patients, as well as individuals with an unknown or undocumented treatment history. Consequently, the denominators for the naïve and experienced subgroups do not mathematically sum to the overall cohort total due to the inclusion of this unstratified patient subset.

**Table 2:** Prevalence and distribution of specific major drug resistance mutations (DRMs) across antiretroviral classes (N = 361).

Mutation	Prevalence, % (n/N), [95% CI]
**PIs**	
V82A	1.11% (4/361), [0.33–2.92]
M46L	0.83% (3/361), [0.17–2.53]
M46I	0.55% (2/361), [0.02–2.13]
**NRTIs**	
M184V	7.48% (27/361), [5.15–10.70]
K65R	5.54% (20/361), [3.57–8.45]
M184I	2.22% (8/361), [1.05–4.39]
Y115F	1.39% (5/361), [0.50–3.30]
D67N	1.11% (4/361), [0.33–2.92]
**NNRTIs**	
G190S	6.65% (24/361), [4.47–9.74]
K101E	4.99% (18/361), [3.13–7.79]
K103N	4.43% (16/361), [2.69–7.13]
Y181C	4.16% (15/361), [2.48–6.80]

## Data Availability

All nucleotide sequences analysed in the study and their associated data have been deposited in the Los Alamos National Laboratory HIV Sequence Database (accession numbers: PX796826-PX797217; PZ684872-PZ685103, PZ685110-PZ685115, PZ685121-PZ685279, PZ685281, PZ685293-PZ685295, PZ685297-PZ685300, PZ685302-PZ685307, PZ685309, PZ685312-PZ685313, PZ685315, PZ685319, and PZ685323-PZ685325).

## Supplementary Materials

The following supporting information can be downloaded at: https://www.mdpi.com/article/10.3390/v18080856/s1; Figure S1: The results of phylogenetic analysis of the studied nucleotide pol sequences (n = 361). The differently colored areas mark HIV-1 clusters depending on their genetic variant: pink marks the HIV-1 sub-subtype A6 cluster, blue marks B, purple marks CRF03_A6B and light green marks CRF63_02A6; Figure S2: Distribution of patient proportions depending on levels of resistance to drugs of different classes of ART.
