# Supplementary material for: Cross-Border Circulation and Molecular Surveillance of HIV-1 in the Azov and Donbas Regions: A Study of Genetic Diversity and Drug Resistance
Source: Viruses. 2026 Aug 5;18(8):856. doi: 10.3390/v18080856 (PMC13517423; doi:10.3390/v18080856)
Supplement: Supplementary file 1 [file viruses-18-00856-s001.zip › viruses-4439835-supplementary.pdf]

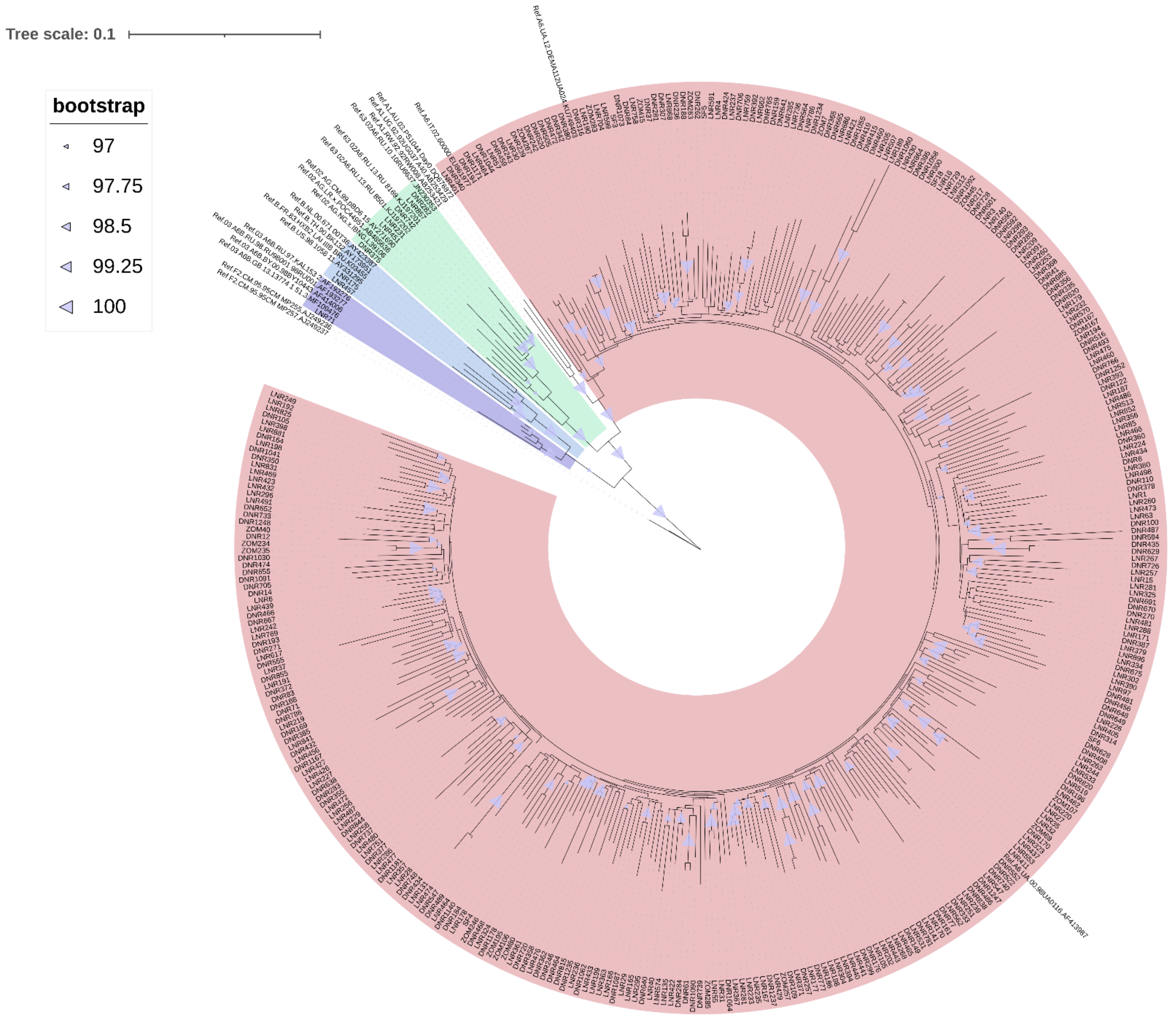

Figure S1. The results of phylogenetic analysis of the studied nucleotide *pol* sequences (n = 361). The differently colored areas mark HIV-1 clusters depending on their genetic variant: pink marks the HIV-1 sub-subtype A6 cluster, blue marks B, purple marks CRF03_A6B and light green marks CRF63_02A6.

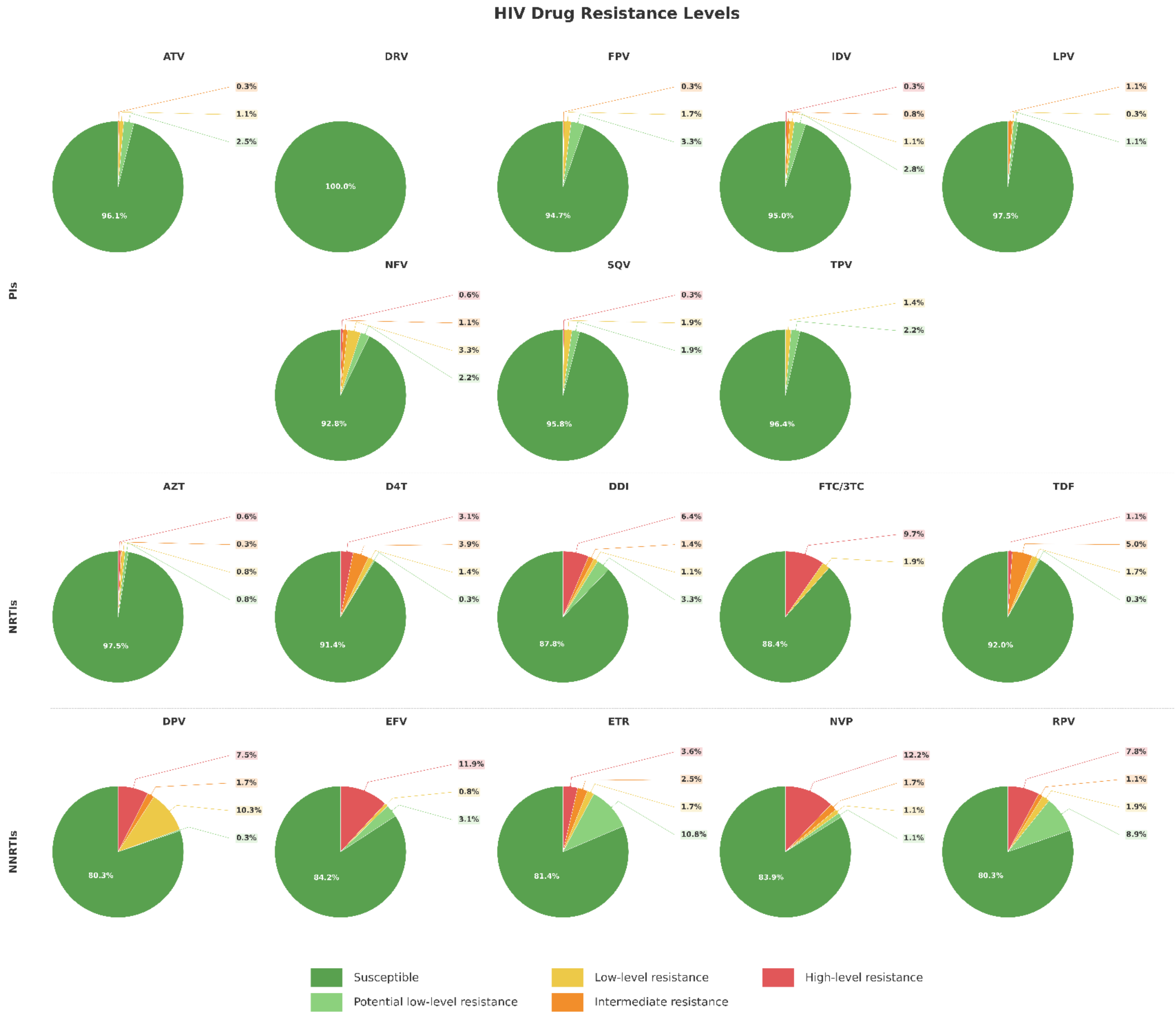

Figure S2. Distribution of patient proportions depending on levels of resistance to drugs of different classes of ART.

Protease Inhibitors (PIs): Over 95% of isolates demonstrated full susceptibility to most PIs, except for (FPV) – 94.74% (342/361; 95% CI: 91.88–96.65) and NFV – 92.80% (335/361; 95% CI: 89.62–95.07). For FPV, potential low-level resistance was identified in 3.32% (12/361; 95% CI: 1.85–5.78), low-level in 1.66% (6/361; 95% CI: 0.68–3.67), and intermediate resistance in 0.28% (1/361; 95% CI: 0.01–1.71). For NFV, potential low-level resistance was 2.22% (8/361; 95% CI: 1.05–4.39), low-level was 3.60% (13/361; 95% CI: 2.06–6.12), intermediate was 1.11% (4/361; 95% CI: 0.33–2.92), and high-level resistance was 0.55% (2/361; 95% CI: 0.02–2.13).

Nucleoside Reverse Transcriptase Inhibitors (NRTIs): Only AZT maintained a susceptibility rate exceeding 95% – 97.51% (352/361; 95% CI: 95.26–98.76). Full susceptibility for other NRTIs was lower: TDF at 91.97% (332/361; 95% CI: 88.67–94.38), D4T at 91.41% (330/361; 95% CI: 88.04–93.92), FTC/3TC at 88.37% (319/361; 95% CI: 84.62–91.30), and DDI at 87.81% (317/361; 95% CI: 84.01–90.82). High-level resistance was documented in 9.70% (35/361; 95% CI: 7.03–13.21) for FTC/3TC and 6.37% (23/361; 95% CI: 4.24–9.42) for DDI.

Non-Nucleoside Reverse Transcriptase Inhibitors (NNRTIs): Susceptibility rates for all NNRTIs were below 85%: EFV – 84.21% (304/361; 95% CI: 80.07–87.63), NVP – 83.93% (303/361; 95% CI: 79.77–87.38), ETR – 81.44% (294/361; 95% CI: 77.09–85.13), DPV – 80.33% (290/361; 95% CI: 75.91–84.12), and RPV – 80.33% (290/361; 95% CI: 75.91–84.12). High-level resistance was more pronounced among first-generation NNRTIs: NVP – 12.19% (44/361; 95% CI: 9.18–15.99); EFV – 11.91% (43/361; 95% CI: 8.94–15.68) than next-generation agents RPV – 7.76% (28/361; 95% CI: 5.39–11.02) and DPV – 7.48% (27/361; 95% CI: 5.15–10.70).
